# Letter to editor regarding the systematic review and meta‐analysis by Coleman et al. on the effectiveness of MF59‐adjuvanted seasonal influenza vaccine in older adults

**DOI:** 10.1111/irv.12891

**Published:** 2021-08-02

**Authors:** J. Kevin Yin, Sandrine I. Samson, Joshua Nealon

**Affiliations:** ^1^ Global Medical Sanofi Pasteur Singapore; ^2^ The University of Sydney Camperdown NSW Australia; ^3^ Global Medical Sanofi Pasteur Lyon France

## AUTHOR CONTRIBUTIONS

**J. Kevin Yin:** Conceptualization; data curation; formal analysis; investigation; methodology. **Sandrine Samson:** Conceptualization; data curation; formal analysis; investigation; methodology. **Joshua Nealon:** Conceptualization; data curation; formal analysis; investigation; methodology.

### PEER REVIEW

The peer review history for this article is available at https://publons.com/publon/10.1111/irv.12891.

Dear Professor Cowling,

We read with interest the meta‐analysis by Coleman et al. published in May 2021 that aimed to determine the effectiveness of seasonal MF59‐adjuvanted influenza vaccine in older adults, finding the adjuvanted vaccine “*was effective in preventing influenza in adults 65 years of age or older*” and, “*compared to standard‐dose egg‐based QIV and TIV, aTIV was significantly more effective in preventing influenza‐related medical encounters*.”^1^ Although we agree with the aim, we suggest that additional explanation of the rationale for study inclusion and discussion of the results in the context of all available evidence would have provided additional clinical and policy perspective.

Meta‐analysis is a useful tool to synthesize information, improve precision and address conflicting claims. Conduct guidelines exist to help guarantee quality, transparency and reproducibility.^2^ Coleman et al. adhered to many of these guidelines: the protocol was pre‐registered; the intervention, comparators and outcomes of interest were described; the risk of bias was assessed using a recognized scale; and standard analytical approaches were used. Despite these methodological strengths, we believe that the strategy for selecting eligible studies may have influenced the conclusions and that the review could have been strengthened in three main areas.

First, the review should have included all relevant studies performed in the indicated timeframe, including randomized control trials (RCTs), which were ineligible. This is important because RCTs are relatively unbiased, provide the highest level of evidence, and are therefore the preferred design for evaluating healthcare interventions to inform clinical and public health decisions.^3^ An RCT comparing MF59‐adjuvanted influenza vaccine with a non‐influenza vaccine comparator in older adults has been recently conducted and was presented at Options X conference in 2019^4^ and published in *Lancet Infectious Diseases* in February 2021.^5^ This trial did not meet the pre‐specified primary endpoint of prevention of RT‐PCR‐confirmed influenza (efficacy vs. non‐influenza comparator 19.8%, 95% CI: −5.8; 38.9). Discussing the discrepancy between this RCT result and the conclusions of this review may have helped readers to understand factors contributing to the apparent conflict.

Second, weighting or stratification by study quality would have provided additional information on the robustness of the review. This is particularly important because observational studies assessing influenza vaccine effectiveness are inherently biased and sensitive to design choices,^6^ all included studies had a moderate/serious risk of bias, and the robustness of a meta‐analysis relies on the quality of the studies included.^7^ At least three included studies have serious overall risk of bias as assessed by the authors or the European Centre for Disease Prevention and Control (ECDC):^8^ two have serious risk of confounding;^9^, ^10^ and one has serious risk of selection bias;^11^ but these were included nonetheless. The review incorporated non peer‐reviewed data, including a 2013 poster^12^ that dominated the effect of pooled estimate (61.4% weigh; Figure 2.A.) but is unpublished and remains publicly unavailable. The resulting estimates exhibited high heterogeneity (*I* ^*2*^ ≥ 94.5% in half of the pooled results) which was discussed at length by the authors, but the combination of low study quality and high variability in observed effects detracts from the value of this analysis to inform clinical practice and policy recommendations.^13^

Finally, it would have been helpful to position the observed results within the context of conflicting, independent evaluations of influenza vaccine performance. Such assessments (using GRADE or similar frameworks) were conducted by the ECDC in 2020,^8^ by Canada's National Advisory Committee on Immunization in 2018,^14^ and more recently by the German Standing Committee on Vaccination.^15^ For example, the ECDC review stated in the discussion summary that “*Overall, there is an absence of high‐quality evidence regarding the efficacy of MF59^®^ adjuvanted influenza vaccines. While data show that they are generally more effective than no vaccination in reducing the risk of laboratory‐confirmed influenza and additional proxy outcomes, their effectiveness compared with traditional vaccine comparators is uncertain and based on limited data*.”^8^

In transparency, the authors of this letter work for a competing vaccine manufacturer and your readers should consider our biases when forming their opinions. However, we believe those opinions should be based on assessment of all available evidence and its quality and certainly including pivotal data from RCTs when they are available.
